# Oral delivery of peptides and proteins: pharmacokinetic boundaries, negative selection, and route triage

**DOI:** 10.3389/fddev.2026.1776167

**Published:** 2026-03-20

**Authors:** Sarfaraz K. Niazi

**Affiliations:** College of Pharmacy, University of Illinois, Chicago, IL, United States

**Keywords:** bioavailability, elimination half-life, ingestible injection devices, macrocyclic peptides, MK-0616, negative selection, octreotide, oral peptide delivery

## Abstract

Oral delivery of peptides and proteins has been pursued for over a century due to its noninvasive nature. Despite sustained innovation across multiple technological generations—such as enteric coatings, enzyme inhibitors, permeation enhancers, nanoparticles, and ingestible devices—most oral peptide programs have failed to achieve regulatory or commercial success. The recent approval of oral semaglutide has renewed enthusiasm but introduced a substantial risk of conceptual over-extrapolation. This review critically examines the pharmacokinetic, pharmacodynamic, regulatory, and economic foundations of oral peptide delivery. Three recurrent failure modes explain the historical record: (1) exposure infeasibility when low bioavailability combines with short elimination half-life, preventing accumulation regardless of formulation sophistication; (2) variability-driven regulatory failure when coefficients of variation exceed thresholds compatible with bioequivalence requirements; and (3) dose escalation leading to gastrointestinal toxicity and prohibitive manufacturing costs before therapeutic efficacy is achieved. Oral semaglutide is analyzed as a boundary case rather than a platform validation. Its success reflects a rare alignment of properties: an exceptionally long half-life (∼168 h), high potency, wide therapeutic window, and time-integrated pharmacodynamics that accommodate low and variable absorption. Most peptides lack this constellation. Oral octreotide, approved under restricted maintenance-only labeling, is another boundary case that illustrates these constraints. Emerging macrocyclic peptides (MK-0616, JNJ-77242113) and small molecule GLP-1 agonists (orforglipron) may reshape the competitive landscape. To prevent predictable failure, this review proposes a negative-selection framework that identifies peptides unsuitable for classical oral delivery at an early stage. For excluded molecules, a route-triage strategy directs candidates toward pulmonary, nasal, buccal, or long-acting injectable delivery. Device-mediated swallowed injection systems differ from oral absorption and are evaluated as combination products governed by device reliability rather than pharmacokinetic enhancement. The fundamental conclusion is that the success of oral delivery depends on molecular pharmacology, not on formulation technology. Oral delivery should be pursued when pharmacology permits and abandoned when it does not—a prerequisite for sustainable advancement in the field.

## 1 Introduction: the persistence of an elusive goal

Oral delivery of peptide and protein therapeutics has been pursued for more than a century, motivated by the practical advantages of noninvasive administration, including convenience, improved patient preference, and the potential for enhanced adherence in chronic diseases (Fosgerau and Hoffmann, 2015; Lau and Dunn, 2018). These advantages are particularly compelling for lifelong therapies such as diabetes, obesity, endocrine disorders, and inflammatory diseases, where cumulative injection burden can negatively affect treatment persistence and quality of life. However, it is essential to distinguish logistical and behavioral advantages from intrinsic clinical superiority. Noninvasive routes do not inherently improve pharmacodynamic precision, therapeutic index, or safety relative to parenteral administration, and conflating convenience with clinical benefit risks obscuring the biological constraints that govern treatment success.

Despite sustained scientific effort, the historical record of the development of oral peptides and proteins is dominated by failure rather than success. Multiple technological generations—including enteric coatings, protease inhibitors, permeation enhancers, lipid-based systems, polymeric nanoparticles, and mucoadhesive formulations—have been developed to address gastrointestinal degradation and epithelial impermeability, yet none have reliably produced predictable systemic exposure suitable for regulatory approval (Morishita and Peppas, 2006; Brayden et al., 2020). These failures have occurred across diverse peptide classes and delivery platforms, suggesting that the primary barrier is not insufficient formulation ingenuity but a fundamental mismatch between peptide pharmacology and the constraints imposed by the oral route.

The recent approval of oral semaglutide represents the first unequivocal demonstration that a systemically acting peptide can be administered orally at a commercial scale with reproducible clinical benefit (Davies et al., 2017; Aroda et al., 2019). This achievement has appropriately renewed interest in oral peptide delivery but has also created a risk of conceptual overgeneralization. Semaglutide’s success has frequently been interpreted as evidence that oral delivery of peptides is now broadly feasible, when in fact it may represent a highly constrained exception that defines the outer boundary of feasibility rather than opening a generalizable pathway forward (Drucker, 2020). Industry scientists are well aware of these constraints, and this review aims to consolidate that understanding into a systematic framework.

Historically, nearly every major therapeutic peptide class has been evaluated for oral delivery. Insulin, calcitonin, parathyroid hormone fragments, growth hormone, glucagon, and vasopressin analogs have advanced through extensive preclinical development and, in many cases, Phase II and Phase III clinical trials (Arbit and Kidron, 2009; Karsdal et al., 2010; Fonte et al., 2013). Although some programs demonstrated transient pharmacodynamic activity, none achieved the combination of predictable exposure, acceptable variability, long-term safety, and economic sustainability required for regulatory approval and widespread clinical use. The cumulative financial investment in these failed efforts amounts to many billions of dollars.

The persistence of failure suggests that the primary obstacle is not the absence of sufficiently sophisticated delivery technologies, but rather a fundamental mismatch between the pharmacokinetic and pharmacodynamic properties of most peptides and the constraints imposed by oral administration. Three interrelated factors dominate outcomes: extremely low and variable oral bioavailability, a short systemic elimination half-life, and high-dose sensitivity with narrow therapeutic windows. When these factors coexist, oral delivery becomes not merely difficult but intrinsically untenable.

The success of oral semaglutide does not invalidate this conclusion; instead, it reinforces it. Semaglutide succeeds precisely because it violates the typical assumptions of peptide pharmacology. Its elimination half-life is extraordinarily long (∼168 h), its potency is exceptionally high, its therapeutic window is wide, and its clinical effects are driven by time-integrated exposure rather than peak concentration. The molecular design principles that enable this extended half-life—including the C18 fatty acid chain, which confers strong albumin binding, and amino acid substitutions that confer DPP-4 resistance—were elucidated during the development of semaglutide (Lau et al., 2015). These properties collectively allow semaglutide to accommodate the extreme variability and low absolute bioavailability inherent to oral peptide absorption. Most peptides and essentially all proteins do not share this constellation of properties.

This review advances the argument that progress in oral peptide and protein therapeutics requires a conceptual shift from platform-driven optimism to pharmacology-driven exclusion. By identifying exclusion criteria rooted in elimination kinetics, dose sensitivity, safety margins, bioequivalence requirements, and cost realities, development programs can be rationally triaged at an early stage. Sophisticated sponsors already apply similar reasoning implicitly; this framework aims to formalize and systematize criteria that experienced developers intuitively understand.

The objectives of this review are therefore fourfold. First, to examine the mechanistic reasons underlying repeated failures in oral peptide and protein delivery. Second, to present a quantitative pharmacokinetic framework demonstrating why short half-life, dose-critical molecules cannot succeed orally, regardless of formulation advances. Third, to propose a negative-selection framework that clearly delineates which peptides and proteins should not be developed as oral dosage forms, using oral semaglutide and oral octreotide as boundary cases rather than templates. Fourth, to survey realistic alternative routes for peptides and proteins unsuitable for oral delivery, including device-mediated swallowed injection approaches. By doing so, this article aims to provide a useful reference framework for development decisions. This analysis addresses peptides and proteins intended for systemic action; local-acting gastrointestinal peptides, where therapeutic effects are exerted within the gut lumen, represent a fundamentally different problem and are not addressed here.

## 2 Mechanistic foundations of historical failure

The pursuit of oral peptide delivery began almost immediately after the therapeutic potential of peptides was first recognized. The discovery of insulin in 1921 transformed diabetes from a fatal disease into a manageable condition, but it also exposed the inconvenience and limitations of parenteral administration for chronic therapy. Within months of insulin’s clinical introduction, investigators began experimenting with oral dosing, often administering vast quantities of crude pancreatic extracts to replicate the glucose-lowering effects observed with injection (Bliss, 1982). These early efforts produced occasional, inconsistent reductions in blood glucose, but they were accompanied by extreme variability and frequent failure, foreshadowing problems that would persist for the next hundred years.

During the pioneering era from the 1920s through the 1950s, oral peptide delivery was pursued primarily through empirical experimentation. Investigators attempted to shield peptides from gastric degradation by coating them with shellac, keratin, gelatin, and cellulose derivatives, reasoning that protection from stomach acid would permit intestinal absorption. While these enteric strategies successfully delayed dissolution, they did not improve systemic exposure. Insulin, once released into the intestine, was rapidly degraded by pancreatic proteases and brush-border enzymes, leaving negligible intact drug available for absorption (Hamman et al., 2005). The failure of these approaches established an early but often overlooked lesson: protection from acid alone is insufficient when enzymatic and permeability barriers remain intact.

### 2.1 The enzymatic barrier

By the 1960s and 1970s, advances in enzymology and gastrointestinal physiology began to clarify the mechanistic basis for these failures. Detailed mapping of proteolytic pathways revealed that peptides undergo a coordinated cascade of degradation, starting with pepsin in the stomach, followed by trypsin, chymotrypsin, and elastase in the small intestine, and culminating in peptidases located on the epithelial brush border and within enterocytes (Morishita and Peppas, 2006). Experimental studies demonstrated that many peptides, including insulin and calcitonin, exhibit intestinal half-lives measured in minutes rather than hours (Mahato et al., 2003). Even when enzymatic degradation was partially inhibited, absorption across the epithelial barrier remained vanishingly small.

### 2.2 The permeability barrier

Concurrently, the epithelial permeability barrier emerged as a second dominant constraint. Tight junctions between intestinal epithelial cells restrict paracellular transport to molecules with radii well below those of therapeutic peptides, while transcellular transport favors lipophilic small molecules rather than hydrophilic macromolecules (Lundquist and Artursson, 2016). Quantitative permeability studies showed that insulin’s effective permeability coefficient was several orders of magnitude below the value required to achieve therapeutic plasma concentrations via oral dosing (Aungst, 2012). These findings established that oral peptide delivery was constrained not by a single barrier but by multiple, overlapping defenses explicitly evolved to prevent systemic absorption of dietary proteins.

### 2.3 The era of permeation enhancers

From the 1980s through the early 2000s, research shifted toward chemical permeation enhancers designed to transiently disrupt epithelial barriers. A wide array of surfactants, bile salts, fatty acids, and chelating agents was evaluated for their ability to increase paracellular or transcellular transport (Maher et al., 2016). Medium-chain fatty acids, particularly sodium caprate (C10), have emerged as the most clinically validated permeation enhancers. Current mechanistic understanding emphasizes membrane fluidization and surfactant effects as primary mechanisms at the 180–500 mg doses used in tablet formulations, rather than tight junction modulation as previously emphasized (Maher et al., 2009; Maher et al., 2016). C10 is the permeation enhancer in MK-0616, currently under NDA review at the FDA, demonstrating its clinical viability at these concentrations.

Salcaprozate sodium (SNAC) represents a distinct enhancer class with a distinct mechanism: local buffering of gastric pH, protection against pepsin-mediated degradation, and facilitation of transcellular transport. SNAC at 300 mg is the enhancer in all approved oral semaglutide formulations (Rybelsus 3/7/14 mg for diabetes, Wegovy 25 mg for obesity). Historical experience with permeation enhancers at high concentrations caused mucosal damage in in vitro and *ex vivo* studies (Whitehead et al., 2008; Maher et al., 2009). However, the two most clinically evaluated enhancers, sodium caprate (C10) at the concentrations used in MK-0616 (180 mg) and SNAC at 300 mg in oral semaglutide formulations, have demonstrated acceptable tolerability profiles in Phase III trials and in marketed products, respectively. To our knowledge, no evidence of barrier compromise or increased pathogen entry has been reported in clinical trials or marketed products using these enhancers. The gastrointestinal adverse events observed with oral semaglutide (nausea, vomiting) are class effects of GLP-1 receptor agonism, not enhancer-related toxicity.

### 2.4 The promise and disappointment of nanotechnology

The emergence of nanotechnology in the 1990s introduced a new wave of optimism. Polymeric nanoparticles, liposomes, solid lipid nanoparticles, and self-emulsifying systems were developed to encapsulate peptides, protect them from enzymatic degradation, and promote epithelial uptake (Des Rieux et al., 2006; Niu et al., 2016). Preclinical studies frequently demonstrated improved stability and modest increases in absorption in rodents. However, translation into humans proved disappointing. The gastrointestinal environment in humans differed sufficiently from animal models to negate many of the observed benefits, and manufacturing challenges, batch-to-batch variability, and regulatory uncertainty further impeded development (Ensign et al., 2012). By the late 2000s, it had become clear that nanocarriers did not fundamentally overcome the absorption barrier for systemically acting peptides.

### 2.5 Device-based approaches and the turn toward bypass

Device-based approaches represented the most ambitious attempts to bypass biological barriers altogether. Ingestible devices capable of deploying microneedles into the intestinal wall, adhering to the epithelium, or delivering peptides via localized injection were proposed as radical solutions (Abramson et al., 2019). While these technologies demonstrated proof-of-concept delivery in controlled experimental settings, they introduced new safety, tolerability, and manufacturing challenges. Chronic use raised concerns regarding mucosal injury, device retention, and patient acceptance, while the complexity and cost of these systems undermined any advantage over conventional injectable therapies (Anselmo and Mitragotri, 2019). These approaches are discussed in detail in Section 9.

### 2.6 The pattern of failure, publication bias, and emerging macrocyclic candidates

Across these successive technological waves, a consistent pattern emerged. When oral peptide programs advanced into clinical development, they encountered one or more insurmountable obstacles: extreme inter- and intra-subject variability, inability to meet bioequivalence criteria, unacceptable local gastrointestinal class effects at effective doses, or prohibitive cost of goods due to dose escalation (Karsdal et al., 2010; Fonte et al., 2013; Brayden et al., 2020). High-profile failures of oral insulin programs, oral calcitonin, oral parathyroid hormone fragments, and oral growth hormone reinforced the same conclusion repeatedly. However, development efforts continued to cycle through new platforms rather than re-evaluating underlying pharmacological assumptions.

An important caveat regarding the evidence base deserves acknowledgment: the oral peptide literature is subject to substantial publication bias. Adverse clinical outcomes and discontinued programs are systematically underreported because sponsors have limited incentive to publish failed studies, and journals favor positive results. Consequently, the actual failure rate of oral peptide programs is likely even higher than the published literature suggests. Many programs that quietly discontinued after Phase I or Phase II without achieving proof of concept are not represented in publicly accessible databases. This publication bias underscores the importance of the negative-selection framework proposed in this review: relying solely on published successes and near-successes provides an incomplete, overly optimistic picture of the feasibility of oral peptides.

Recent advances in macrocyclic peptide design have produced a new generation of oral candidates that warrant attention. MK-0616, an oral macrocyclic peptide PCSK9 inhibitor developed by Merck, demonstrated robust LDL-C reductions of up to 60.9% in Phase 2b trials and is now under NDA review (Ballantyne et al., 2023). Notably, MK-0616 is formulated with sodium caprate (C10) as a permeation enhancer. JNJ-77242113, an oral IL-23 inhibitor macrocycle for psoriasis, and Luna-18, an oral macrocyclic peptide for metabolic indications, represent additional examples of this emerging class. These macrocyclic peptides share key design features: constrained conformations that confer proteolytic stability, optimized lipophilicity for membrane permeability, and molecular weights intermediate between traditional linear peptides and proteins. While they are subject to the same pharmacokinetic constraints described in this review, their enhanced stability and engineered permeability may expand the boundary of oral feasibility beyond what was achievable with earlier peptide candidates. Importantly, all successful examples to date exhibit relatively long half-lives or AUC-driven pharmacodynamics, consistent with the framework presented here.

The historical record, therefore, offers a clear but frequently ignored lesson. Oral peptide and protein delivery has not failed for lack of ingenuity or persistence, but because most peptides and proteins possess pharmacokinetic and pharmacodynamic properties fundamentally incompatible with the constraints of the oral route. The repeated reinvention of delivery technologies has obscured this reality, allowing optimism to persist even as evidence accumulated against general feasibility. Understanding this history is essential, not as a chronicle of technological shortcomings, but as a foundation for identifying which molecules should be excluded from oral development before resources are irreversibly committed.

## 3 The pharmacokinetic feasibility gate: exposure, half-life, and the accumulation requirement

The central determinant of whether an orally administered peptide achieves therapeutic systemic exposure is not the sophistication of the delivery platform, but the relationship among absorption rate, elimination half-life, and dosing interval. While decades of research have focused on improving epithelial permeability and protecting peptides from enzymatic degradation, comparatively little attention has been paid to the arithmetic of accumulation at steady state. This imbalance has led to repeated attempts to solve an insoluble problem with increasingly complex technologies.

### 3.1 The bioavailability constraint

The oral bioavailability of peptides intended for systemic action is typically less than 1%, even under optimized conditions (Morishita and Peppas, 2006; Maher and Brayden, 2021). This low fraction reflects the combined effects of luminal degradation, limited permeability, and first-pass metabolism. According to the FDA Clinical Pharmacology Review for Rybelsus (oral semaglutide), absolute bioavailability is approximately 0.4%–1% when administered under strictly controlled conditions: with no more than 4 oz of water, on an empty stomach, and followed by a 30-min post-dose fast before food or other medications (FDA, 2019). These values align with widely cited estimates in the literature and provide a realistic baseline for oral peptide absorption.

Importantly, all current oral peptide formulations require fasting conditions; administration with food results in near-zero bioavailability. This represents a fundamental limitation that small molecule alternatives like orforglipron circumvent. Low bioavailability alone does not preclude efficacy—many small-molecule drugs with low bioavailability are clinically effective. What distinguishes peptides is that low bioavailability is almost invariably accompanied by rapid systemic clearance and, in many cases, narrow therapeutic windows.

### 3.2 The accumulation principle and feasibility inequality

At steady state, the amount of drug present in the body is governed by the balance between input and elimination. For drugs administered at regular intervals, accumulation depends on the elimination rate constant and the dosing interval. When the elimination half-life is much shorter than the dosing interval, accumulation is negligible, and steady-state exposure approximates single-dose exposure (Rowland and Tozer, 2011; Gibaldi and Perrier, 1982). Under these conditions, increasing dose size does not meaningfully increase average systemic exposure; it merely increases transient luminal concentrations.

The accumulation factor (R) for repeated dosing at a fixed interval can be derived from standard pharmacokinetic theory and approximated as:

$$R=1\;/\;\left(1-e^{\left(-k\times \tau \right)}\right)$$

Where k is the elimination rate constant (k = 0.693/t½) and τ is the dosing interval. This relationship is established in foundational pharmacokinetic texts (Rowland and Tozer, 2011; Gibaldi and Perrier, 1982). When t½ << τ (half-life much shorter than dosing interval), the exponential term approaches zero and R approaches 1, meaning no accumulation occurs. When t½ >> τ (half-life much longer than dosing interval), R becomes large, permitting substantial accumulation despite low single-dose absorption.

A common misconception is that long half-life drugs would accumulate indefinitely. In reality, steady state is reached when the rate of drug input equals the rate of elimination, which occurs after approximately 4–5 half-lives. For semaglutide (∼168 h half-life), steady state is achieved after approximately 4–5 weeks of daily dosing. At this point, the amount eliminated each day equals the amount absorbed, and plasma concentrations plateau. The accumulation factor (R ≈ 10–15× for semaglutide with once-daily dosing) describes the ratio of steady-state exposure to single-dose exposure, not a process of unlimited accumulation. This pharmacokinetic principle is described in standard texts (Rowland and Tozer, 2011).

For oral feasibility with once-daily dosing (τ = 24 h), the following conditions must simultaneously hold: (1) Bioavailability (F): Typically, <1% for peptides under optimal conditions. (2) Elimination half-life (t½): Must be sufficiently long (≥24 h as a practical minimum threshold) that accumulation occurs over multiple doses. (3) Potency: Must be high enough that the small, absorbed dose produces a pharmacological effect. (4) Therapeutic window: Must be sufficiently broad to accommodate the variability inherent in oral absorption.

### 3.3 Why elimination half-life is the gatekeeper

Most therapeutic peptides exhibit elimination half-lives measured in minutes to a few hours. Examples include glucagon (minutes), calcitonin (∼1 h), parathyroid hormone fragments (minutes), vasopressin analogs (10–35 min), oxytocin (1–6 min), and insulin and its analogs (1–6 h depending on formulation) (Usmani et al., 2017). For such molecules, elimination is effectively complete between daily oral doses. Even if a small fraction of an oral dose enters the systemic circulation, it is cleared long before the next dose is administered.

Consider a peptide with a 2-h half-life administered once daily. After 24 h, only (1/2)^12^ = 0.024% of the absorbed dose remains in the body. Even after weeks of dosing, no clinically meaningful accumulation occurs. For such a peptide, the only way to increase systemic exposure is to increase the amount absorbed per dose—but with bioavailability fixed at <1%, this requires enormous dose escalation with all its attendant problems.

By contrast, semaglutide, with a ∼168-h half-life, accumulates substantially over the first 4–5 weeks of daily dosing. At steady state, body burden is approximately 10–15 times the single-dose absorbed amount, bringing plasma concentrations into the therapeutic range despite extremely low and variable per-dose absorption.

**FIGURE 1:**
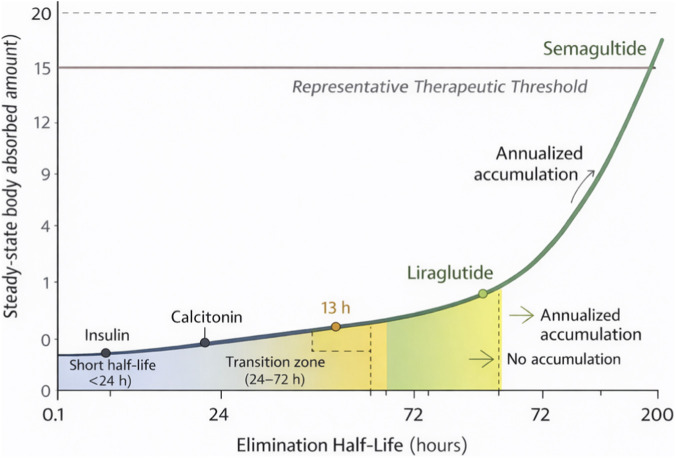
Accumulation Factor (R) at Steady State as a Function of Elimination Half-Life at Low Oral Bioavailability. This figure, generated by the author, illustrates the quantitative relationship between elimination half-life and steady-state accumulation. The x-axis represents elimination half-life in hours (1h, 2h, 6h, 12h, 24h, 48h, 72h, 168 h), and the y-axis represents the Accumulation Factor (R), defined as the ratio of steady-state body burden to single-dose absorbed amount. The curve is calculated using the standard accumulation equation R = 1/(1 - e^(-k × τ)^) where k = 0.693/t½ and τ = 24 h (once-daily dosing). At short half-lives (<6h), R approaches 1.0, indicating negligible accumulation between doses. As half-life increases beyond 24 h, R increases substantially, reaching approximately 10–15× for semaglutide’s ∼168-h half-life. The figure demonstrates that meaningful accumulation (R > 5) requires half-lives of approximately 24 h or more when bioavailability is <1%, explaining why short-half-life peptides cannot achieve therapeutic steady-state concentrations regardless of formulation sophistication. Model assumes standard first-order elimination kinetics (Rowland and Tozer, 2011; Gibaldi and Perrier, 1982).

Figure 1 quantifies this relationship, demonstrating that half-life, not absorption technology, is the dominant determinant of oral feasibility. Oral octreotide (Mycapssa) presents an instructive case that merits specific discussion. While octreotide’s systemic half-life is approximately 1.5 h, the Mycapssa formulation uses a transient permeability enhancer (TPE) technology. The formulation releases both the peptide and the permeation enhancer at high concentration, quickly together in the small intestine following enteric coating dissolution, enabling rapid absorption. More importantly, octreotide was approved only for maintenance therapy in patients already stabilized on injectable octreotide—a deliberately restricted indication that acknowledges the limitations of oral delivery. The high variability (CV 50%–80%) was accepted because: (1) the patient population was already dose-optimized, (2) growth hormone suppression integrates over time (AUC-driven), and (3) the indication did not require treatment-naïve dose finding. This represents a boundary case where regulatory flexibility, combined with restricted labeling, enabled approval despite pharmacokinetic constraints that would preclude approval for most indications (Melmed et al., 2015).

### 3.4 Sensitivity analysis: effects of bioavailability improvements and dosing frequency

A legitimate question arises: could modest improvements in bioavailability or modified dosing strategies overcome the half-life constraint? Sensitivity analysis indicates that the answer is generally no for short-half-life peptides. Consider three scenarios for a peptide with a 2-h half-life:

Scenario 1—Bioavailability improvement from 1% to 3%: This three-fold improvement in bioavailability increases single-dose systemic exposure proportionally but does not alter the fundamental accumulation problem. With a 2-h half-life and once-daily dosing, R ≈ 1.0, meaning steady-state body burden equals single-dose absorption regardless of whether that single dose delivers 1 mg or 3 mg to systemic circulation. The peptide is still cleared before the next dose.

Scenario 2—Twice-daily dosing: Reducing the dosing interval to 12 h modestly increases the accumulation factor. For a 2-h half-life peptide, R increases from ∼1.0 to approximately 1.02—a negligible improvement. Even with four-times-daily dosing (τ = 6 h), R ≈ 1.12. The fundamental mismatch between half-life and dosing interval cannot be overcome by increasing dosing frequency.

Scenario 3—Modified-release oral formulation: Sustained-release oral formulations are theoretically attractive but face insurmountable practical obstacles for peptides. Peptide stability in the gastrointestinal environment is measured in minutes; sustained release over hours would expose the peptide to progressive degradation. Furthermore, the permeability window for peptide absorption is limited to specific intestinal regions; once the dosage form passes these regions, no further absorption occurs, regardless of the drug release rate. The physics of oral peptide delivery fundamentally preclude the sustained-release strategies that succeed with small molecules.

In contrast, consider a peptide with a 168-h half-life (like semaglutide). If bioavailability increases from 1% to 3%, steady-state body burden increases approximately threefold because the high accumulation factor (∼12×) amplifies this increase. For long-half-life peptides, improvements in bioavailability translate directly into therapeutic benefit; for short-half-life peptides, they do not.

### 3.5 Proteins: an even more severe constraint

Proteins face an even more severe constraint. Due to their larger molecular size, proteins exhibit substantially lower intrinsic permeability than peptides. While many proteins have long systemic half-lives when administered parenterally, their oral bioavailability is orders of magnitude lower than that of peptides, often approaching zero (Anselmo and Mitragotri, 2019). Under these conditions, even favorable elimination kinetics cannot overcome the absorption barrier, rendering accumulation negligible regardless of dosing frequency. This is why no systemically acting protein has ever been successfully delivered through classical oral absorption.

The elimination half-life, therefore, serves as a gatekeeper for oral feasibility. Peptides with short half-lives are not merely complex to deliver orally; they are pharmacokinetically incompatible with the oral route. This conclusion follows directly from mass balance and clearance kinetics and does not depend on assumptions about specific delivery technologies. Recognizing this constraint early in development is essential for rational decision-making and for avoiding the repetition of historical failures.

## 4 Variability, dose criticality, and regulatory infeasibility

Beyond pharmacokinetic infeasibility rooted in accumulation failure, oral peptide and protein development encounters a second, distinct barrier at the regulatory interface: the inability to demonstrate reproducible exposure and bioequivalence. Regulatory approval of orally administered drugs requires not only evidence of efficacy and safety, but also assurance that exposure is sufficiently predictable to permit consistent dosing across populations. For most peptides, the intrinsic variability of oral absorption exceeds what regulators can reasonably accept, particularly for dose-critical molecules.

### 4.1 Intrinsic variability of oral peptide absorption

Oral peptide absorption is characterized by extreme inter- and intra-subject variability, with coefficients of variation (CV) frequently exceeding 50%–60% even under controlled fasting conditions (Maher and Brayden, 2021). This variability arises from differences in gastric emptying rate, intestinal transit time, regional enzyme expression, mucosal integrity, individual transporter expression, and dietary state. Unlike small molecules, peptides are detectable only at the margins, rendering them acutely sensitive to physiological fluctuations that would have a negligible impact on conventional drugs.

Food effects represent a particularly intractable source of variability. Absorption enhancers and protective formulations often interact with dietary components, leading to significant differences between fed and fasted states. Clinical studies of oral insulin, calcitonin, and other peptides consistently showed marked reductions or unpredictable changes in exposure when administered with food, necessitating strict fasting requirements that proved impractical for chronic therapy (Hamman et al., 2005; Arbit and Kidron, 2009). Oral semaglutide requires administration under strictly controlled conditions—no more than 4 oz of water, an empty stomach, and a 30-min post-dose fast—to achieve reproducible absorption (FDA, 2019). These requirements are manageable for a once-daily medication but illustrate the sensitivity of peptide absorption to ambient conditions.

### 4.2 Two classes of variability accommodation: why the distinction matters

Not all variability is equally consequential. The critical distinction—and one that has received insufficient attention in the oral peptide literature—is between molecules that can accommodate high absorption variability and those for which such variability is clinically unacceptable. This distinction also determines whether oral delivery is appropriate for chronic maintenance therapy versus episodic or rescue indications.

Variability-accommodating pharmacodynamics (time-integrated effects): When pharmacodynamic effects integrate over time and depend on the area under the curve (AUC) or sustained receptor occupancy rather than peak concentration, transient fluctuations in plasma concentration have limited clinical consequences. Semaglutide exemplifies this category. Its effects on glycemic control and body weight depend on sustained GLP-1 receptor occupancy over days to weeks, not on achieving specific peak concentrations (Drucker, 2018; Drucker, 2020). A given day’s absorption may be 50% higher or lower than average. Still, because the drug accumulates over weeks and effects integrate over time, these fluctuations are buffered at the pharmacodynamic level. Such molecules are appropriate candidates for chronic maintenance therapy via oral delivery, provided other feasibility criteria are met.

Variability-sensitive pharmacodynamics (peak-driven, dose-critical effects): When effects are peak-driven or the therapeutic window is narrow, variability directly translates into clinical harm. Insulin exemplifies this category. Slight deviations in systemic exposure can precipitate hypoglycemia (if exposure is too high) or loss of glycemic control (if exposure is too low), outcomes that are immediate and potentially life-threatening (Cryer, 2008; Heinemann, 2002). A formulation that produces even a two-fold difference in exposure between doses or between subjects is clinically unacceptable for insulin. Similar considerations apply to glucagon (where precise dosing is needed for hypoglycemia rescue), vasopressin analogs (where water balance effects are immediate), oxytocin (where uterine contractile effects are acute), and PTH (1–34) (where the anabolic effect on bone requires specific pulsatile peaks) (Verbalis, 2003; Arrowsmith and Wray, 2014; Rosen, 2005). These molecules are particularly unsuitable for episodic or rescue indications via oral delivery, where rapid and reliable onset is essential.

### 4.3 Bioequivalence standards and regulatory reality

Bioequivalence standards further compound this problem. Regulatory guidance for orally administered drugs typically requires that the 90% confidence intervals for key pharmacokinetic parameters (Cmax and AUC) fall within predefined limits, commonly 80%–125% of a reference product (FDA, 2003; Rowland and Tozer, 2011). For oral peptides with inherent high variability, meeting these criteria is mathematically improbable without large study populations or restrictive dosing conditions that undermine real-world usability.

Numerous oral peptide programs failed not because efficacy could not be demonstrated in some patients under some conditions, but because variability precluded regulatory acceptance. This represents a distinct failure mode that cannot be addressed solely through formulation optimization. The problem is not that these formulations fail to deliver the drug; instead, they provide drug unpredictably, in a way that regulators—correctly—cannot accept for dose-critical indications (Fonte et al., 2013; Karsdal et al., 2010).

## 5 Dose escalation, gastrointestinal class effects, and economic constraints

**FIGURE 2:**
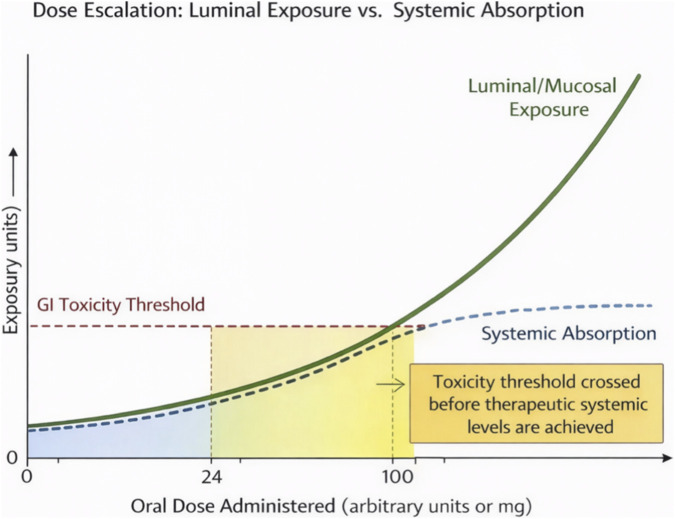
Dose Escalation: Luminal Exposure vs. Systemic Absorption. This author-generated figure schematically illustrates the dissociation between luminal and systemic exposure during dose escalation for oral peptides. As administered dose increases (x-axis), luminal exposure increases linearly while systemic absorption plateaus due to saturation of transport mechanisms and permeability limitations. The toxicity threshold is reached in the luminal compartment before therapeutic systemic levels are achieved, explaining the dose-limiting gastrointestinal adverse events consistently observed in oral peptide programs. The figure demonstrates why dose escalation cannot compensate for low bioavailability in peptide delivery: increasing dose primarily increases local gut exposure rather than systemic exposure, leading to gastrointestinal class effects (nausea, vomiting, diarrhea) at doses below those required for therapeutic efficacy. This phenomenon has been documented in multiple failed oral peptide programs (Karsdal et al., 2010; Fonte et al., 2013).

When the elimination half-life is short and steady-state accumulation is negligible, the most intuitive response in oral peptide and protein development has been dose escalation. The underlying assumption is that if only a small fraction of an oral dose reaches the systemic circulation, increasing the administered dose should proportionally increase exposure. In practice, this strategy has repeatedly failed and constitutes a third, independent failure mode distinct from pharmacokinetic infeasibility and variability-driven regulatory failure. Dose escalation does not circumvent the constraints imposed by low bioavailability and rapid clearance; instead, it disproportionately increases luminal exposure, leading to class-related gastrointestinal effects, unpredictable safety profiles, and economic infeasibility before therapeutic efficacy can be achieved (Figure 2).

### 5.1 Oral semaglutide 25 mg: dose optimization for obesity

The December 2025 FDA approval of oral semaglutide 25 mg (Wegovy) for weight management represents an important development. The OASIS 4 trial demonstrated 16.6% mean weight loss at 64 weeks with adherence, comparable to injectable Wegovy 2.4 mg (Wharton et al., 2025). The higher dose (25 mg vs. 14mg for diabetes) reflects the greater systemic exposure requirements for weight-loss indications. Importantly, the formulation still uses 300 mg SNAC, and the dosing conditions remain similar to Rybelsus.

The higher dose does not necessarily imply improved formulation efficiency; rather, it reflects dose optimization to meet the pharmacodynamic requirements of the obesity indication. The accumulation principle remains unchanged: the ∼168-h half-life permits therapeutic steady-state concentrations despite low per-dose absorption. The approval reinforces that semaglutide’s success depends on its exceptional pharmacokinetic properties rather than a formulation breakthrough.

### 5.2 Economic constraints and cost scaling

Beyond pharmacodynamic considerations, dose escalation imposes profound economic penalties. Manufacturing costs for peptides scale approximately linearly with dose, particularly for synthetic peptides and recombinant proteins produced at commercial scale. Injectable peptides typically require milligram-scale doses with near-complete bioavailability. In contrast, oral formulations with bioavailability below 1% require dose multiplication by factors of 10–100 to deliver equivalent systemic exposure. This multiplication dramatically increases raw material requirements, manufacturing capacity demands, and quality control burdens (Fosgerau and Hoffmann, 2015; Lau and Dunn, 2018).

For example, a peptide requiring 1 mg per day via injection would require approximately 100 mg per day orally at 1% bioavailability. This increase translates into a two-order-of-magnitude rise in annual active pharmaceutical ingredient consumption per patient. For commodity peptides such as insulin, where margins are already constrained, and manufacturing is optimized for injectable use, such increases are incompatible with existing cost structures.

Oral semaglutide represents a rare exception to this pattern. Its weekly-equivalent systemic dose is exceptionally small due to its long elimination half-life, high receptor potency, and time-integrated pharmacodynamics. As a result, even a one-hundred-fold oral dose multiplier remains economically tolerable. This exception reinforces rather than undermines the central conclusion: economic feasibility emerges from molecular pharmacology, not from delivery ingenuity.

## 6 Semaglutide and octreotide as boundary cases: molecular properties and non-transferability

The approval of oral semaglutide represents a singular achievement in the history of peptide therapeutics and warrants careful analysis precisely because it is exceptional. While often cited as evidence that oral peptide delivery has become broadly feasible, a rigorous examination reveals the opposite. Semaglutide succeeds not because the fundamental barriers to oral peptide delivery have been overcome, but because its molecular and pharmacological properties uniquely accommodate those barriers. Understanding semaglutide as a boundary case rather than a platform validation is essential to prevent inappropriate extrapolation and align future development strategies with biological reality. Industry scientists are well aware of these constraints; this section systematizes that understanding.

### 6.1 Enabling properties of semaglutide

**FIGURE 3:**
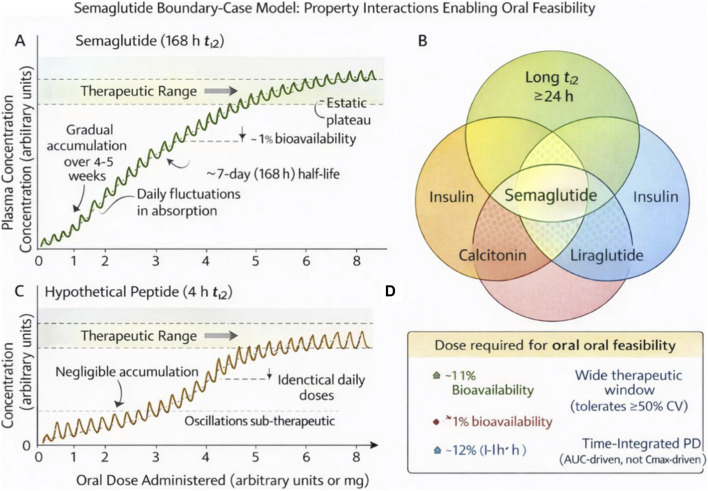
Semaglutide Boundary-Case Model: **(A)** Semaglutide; **(B)** Semaglutide properties; **(C)** Hypothetical peptide; **(D)** Properties of hypothetical peptide. Property Interactions Enabling Oral Feasibility. This author-generated schematic illustrates how semaglutide's four key properties interact to enable oral bioavailability, in contrast to a representative peptide with a short half-life. The model demonstrates that success requires the simultaneous satisfaction of all criteria: a long half-life enables accumulation, high potency permits efficacy at low absorbed doses, a wide therapeutic window tolerates variability, and time-integrated pharmacodynamics buffers day-to-day fluctuations; failure of any single criterion renders oral administration infeasible.

Semaglutide’s feasibility as an oral therapy stems from a deliberate, highly optimized molecular design. Structurally, semaglutide is a glucagon-like peptide-1 receptor agonist engineered to resist rapid enzymatic degradation and to achieve prolonged systemic persistence. Specific amino acid substitutions confer resistance to dipeptidyl peptidase-4, a primary clearance pathway for native GLP-1, while attachment of a C18 fatty acid side chain promotes strong, reversible binding to serum albumin. This albumin association shields the peptide from renal filtration and proteolytic degradation, extending its elimination half-life to approximately 168 h (Knudsen and Lau, 2019). The molecular basis of these modifications and their contribution to pharmacokinetic performance have been described in detail during the discovery and optimization of semaglutide (Lau et al., 2015) (Figure 3).

### 6.2 Clinical pharmacokinetics and formulation requirements

Clinical pharmacokinetic data underscore the fragility of semaglutide’s absorption window. Absolute bioavailability ranges from approximately 0.4–1.0 percent, with substantial inter- and intra-subject variability. Coefficients of variation for maximum concentration and area under the curve commonly exceed thirty to sixty percent (Granhall et al., 2019; FDA, 2019). Strict administration requirements, including fasting conditions, limited water intake, and delayed post-dose food consumption, are necessary to achieve reproducible absorption.

The oral formulation (Rybelsus, Wegovy pill) co-formulates semaglutide with salcaprozate sodium (SNAC, also known as sodium N-[8-(2-hydroxybenzoyl)amino]caprylate). SNAC facilitates absorption through a combination of mechanisms: local pH buffering, protection from pepsin-mediated degradation, and enhancement of transcellular transport (Buckley et al., 2018). Critically, however, SNAC does not fundamentally improve bioavailability into a range that would be viable for peptides lacking semaglutide’s pharmacokinetic properties. It enables absorption at levels that are useful only because semaglutide’s long half-life permits accumulation despite low per-dose uptake.

### 6.3 Near-boundary cases: oral octreotide and other partial successes

Oral octreotide (Mycapssa) represents a partial success that illustrates boundary constraints. The drug was approved only for maintenance therapy in acromegaly patients already stabilized on injectable octreotide—not for treatment-naïve patients. The label acknowledges high variability (CV 50%–80%) and requires strict dosing conditions. This partial success illustrates how peptides at the boundary may achieve approval under restricted indications while still confirming the fundamental constraints (Melmed et al., 2015).

Some oral peptide programs were discontinued not primarily because of biological infeasibility but rather due to strategic portfolio decisions, changes in the competitive landscape, or resource-allocation decisions. However, careful examination of disclosed data typically reveals that variability, modest efficacy relative to injectable comparators, or manufacturing economics played substantial roles in these decisions. True 'strategic-only' discontinuations of biologically successful oral peptide programs are rare in the published record.

Peptides with local-systemic hybrid effects deserve separate consideration. Some peptides exert both local gastrointestinal effects and systemic effects. For such molecules, oral delivery may achieve meaningful therapeutic benefit through local activity even if systemic absorption is inadequate. These cases fall outside the scope of the present analysis, which addresses peptides requiring systemic exposure for efficacy.

### 6.4 Emerging macrocyclic peptides: a new generation of oral candidates

Recent advances in macrocyclic peptide design have produced a new generation of oral candidates that warrant attention. MK-0616, an oral macrocyclic peptide PCSK9 inhibitor developed by Merck, demonstrated robust LDL-C reductions of up to 60.9% in Phase 2b trials and is now under NDA review (Ballantyne et al., 2023). Notably, MK-0616 is formulated with sodium caprate (C10) as a permeation enhancer. The drug’s pharmacokinetic profile supports meaningful systemic exposure with once-daily dosing.

JNJ-77242113, an oral IL-23 inhibitor macrocycle for psoriasis, and Luna-18, an oral macrocyclic peptide for metabolic indications, represent additional examples of this emerging class. These macrocyclic peptides share key design features: constrained conformations that confer proteolytic stability, optimized lipophilicity for membrane permeability, and molecular weights intermediate between traditional linear peptides and proteins.

While these molecules are subject to the same pharmacokinetic constraints described in this review, their enhanced stability and engineered permeability may expand the boundary of oral feasibility beyond what was achievable with earlier peptide candidates. Importantly, all successful examples to date exhibit relatively long half-lives or AUC-driven pharmacodynamics, consistent with the framework presented here.

### 6.5 Small molecule GLP-1 agonists: a paradigm shift?

The development of orforglipron, a small molecule (non-peptide) oral GLP-1 receptor agonist by Eli Lilly, introduces a paradigm-shifting consideration. Orforglipron can be taken at any time, without food or water restrictions, offering substantial practical advantages over peptide formulations that require fasting. Phase 3 trials (ATTAIN-1, ATTAIN-2, ACHIEVE series) demonstrated approximately 12% weight loss and robust glycemic control, with an NDA submitted to the FDA under the Commissioner’s National Priority Voucher program (Eli Lilly and Company, 2025).

If approved, orforglipron would represent direct competition to oral semaglutide, potentially at lower cost and with superior convenience. This development raises a fundamental question: does the future of oral GLP-1 therapy belong to small molecules rather than peptides? The negative-selection framework suggests yes—small molecules escape the pharmacokinetic constraints (proteolytic degradation, low permeability, food effects) that limit oral peptides. The success of oral semaglutide may ultimately be superseded by small-molecule alternatives that offer comparable efficacy without the absorption limitations inherent to peptide delivery.

### 6.6 Why the success is not transferable

Attempts to extrapolate the success of semaglutide to other peptides ignore these interacting requirements. Consider liraglutide, a closely related GLP-1 receptor agonist with substantial structural similarity. Liraglutide has a half-life of approximately 13 hours—about 13-fold shorter than semaglutide’s (Knudsen and Lau, 2019). At this half-life, the accumulation factor for once-daily dosing is approximately 1.5–2×, indicating that the steady-state body burden is only modestly higher than that after a single dose. Combined with lower potency compared to semaglutide, this renders liraglutide far less accommodating of oral variability. An oral liraglutide formulation would need to achieve substantially higher and more consistent bioavailability than oral semaglutide—a requirement that the current state of absorption technology cannot meet.

**FIGURE 4:**
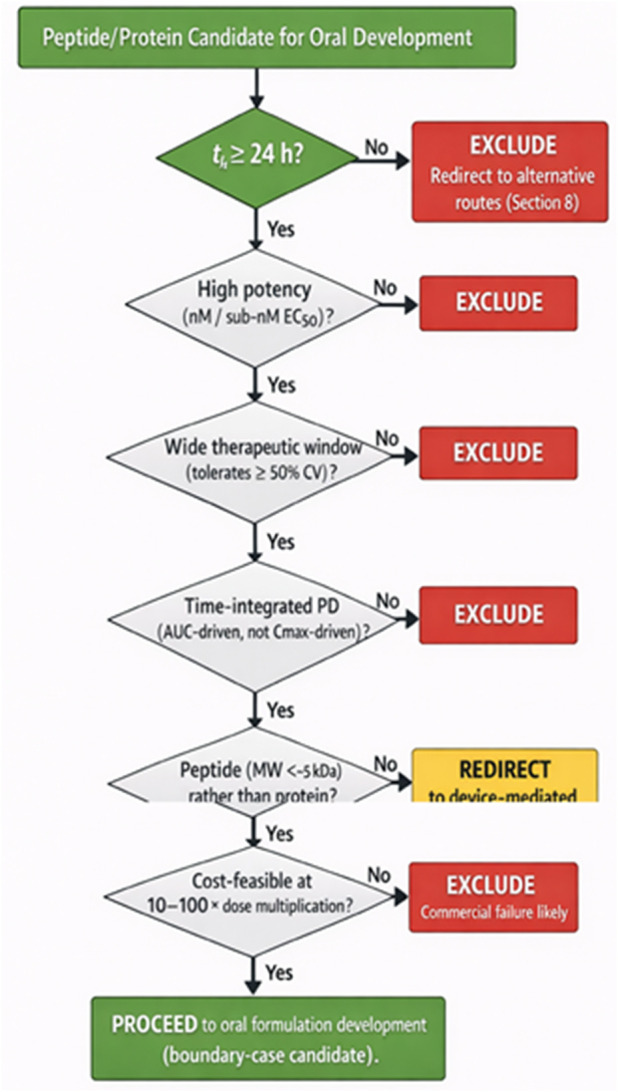
Negative-Selection Decision Flowchart for Oral Peptide and Protein Development. This author-generated flowchart presents the negative-selection framework as a practical decision tool for early-stage development triage. Each decision gate evaluates a specific pharmacological criterion; failure at any gate triggers exclusion from classical oral development and redirection to alternative routes (pulmonary, nasal, buccal, long-acting injectable) or to device-mediated, swallowed-injection delivery. Gate sequence: (1) Elimination half-life ≥24 h? (2) High potency (low nM EC50)? (3) Wide therapeutic window (accommodates ≥50% CV)? (4) AUC-driven pharmacodynamics? (5) Peptide size (<50 amino acids)? (6) Economic feasibility (10–100× dose sustainable)? Molecules that pass all gates may justify investment in oral development. Molecules failing any gate should be redirected to alternative delivery strategies, including injection or device-mediated routes, rather than abandoned. The flowchart consolidates established pharmacokinetic understanding into a systematic decision tool that experienced developers can apply at candidate selection (Rowland and Tozer, 2011; FDA, 2003; DiMasi et al., 2016).

The critical lesson from semaglutide is therefore not that oral peptide delivery has been solved, but that success is contingent on an extraordinary alignment of molecular and pharmacological properties. Semaglutide succeeds because it accommodates low and variable absorption, not because absorption has been fundamentally improved. Treating this outcome as platform validation invites repetition of historical errors and misallocation of development resources. Instead, semaglutide should be viewed as a boundary case that delineates the conditions under which oral delivery is possible and, more importantly, those under which it is not. The properties that enable semaglutide’s success are intrinsic to the molecule and cannot be conferred by formulation technology on molecules that lack them (Figure 4).

## 7 The negative-selection framework: a decision logic for early triage

The cumulative evidence presented in the preceding sections supports a clear conclusion: the feasibility of oral delivery of peptides and proteins is constrained by biological factors in pharmacokinetics, safety, regulatory requirements, and economics. While much of the historical literature has focused on identifying enabling technologies, a more productive approach is to define exclusion criteria that identify molecules for which oral development is inherently unsuitable. This negative-selection framework consolidates established understanding into a systematic decision tool, shifting decision-making upstream and allowing sponsors and regulators to triage candidates before committing substantial resources to development paths destined to fail. Sophisticated sponsors already apply similar reasoning implicitly; this framework aims to formalize and systematize criteria that experienced developers intuitively understand (Table 1).

**TABLE 1:** Negative-selection decision matrix for oral peptide and protein development.

Decision gate	Evaluation question	If yes	If no	Mechanistic rationale
1. Elimination Half-Life	Is systemic t½ ≥ 24 h?^a^	Proceed to Gate 2	EXCLUDE from oral	Short t½ prevents accumulation at low F (Rowland and Tozer, 2011; Gibaldi and Perrier, 1982)
2. Potency	Is the peptide highly potent (low nM EC50)?	Proceed to Gate 3	EXCLUDE from oral	Low potency cannot achieve efficacy at <1% absorbed dose
3. Therapeutic Window	Is window wide enough to accommodate ≥50% CV?	Proceed to Gate 4	EXCLUDE from oral	Narrow window magnifies risk from variable absorption (FDA, 2003)
4. Pharmacodynamic Driver	Is efficacy driven by AUC rather than Cmax?	Proceed to Gate 5	EXCLUDE from oral	Peak-dependent effects incompatible with oral variability (Verbalis, 2003)
5. Molecular Size	Is molecule a peptide (<∼50 AA)?	Proceed to Gate 6	REDIRECT to device	Proteins have near-zero epithelial permeability
6. Economic Feasibility	Can 10–100× dose be sustained economically?	May justify oral development	EXCLUDE: commercial failure likely	Manufacturing costs scale linearly with dose (DiMasi et al., 2016)

^a^ The 24-h threshold represents a practical minimum for once-daily dosing with meaningful accumulation. Peptides with half-lives of 12–24 h may be viable with twice-daily dosing or if other enabling properties (very high potency, very wide therapeutic window) partially compensate. Successful examples include semaglutide (∼168h), an oral octreotide formulation (restricted maintenance indication), and emerging candidates such as I-338 insulin (∼60h), which used sodium caprate (C10) in Novo Nordisk’s Phase II, trials (Halberg et al., 2019).

### 7.1 Application to FDA-approved and emerging peptides

Table 2 applies this framework to representative FDA-approved peptides and emerging candidates, demonstrating how few molecules meet the criteria for oral feasibility and why the historical record of failure is the expected outcome rather than a surprising result. Table 3 represents the current regulatory framework.

**TABLE 2:** Peptides classified by oral delivery feasibility.

Peptide	t½	Dose sensitivity	PD Driver	Oral feasibility	Framework analysis
Semaglutide	∼168 h	Low	AUC/time-integrated	APPROVED (boundary)	Passes all gates (Lau et al., 2015)
Octreotide (Mycapssa)	∼1.5 h^a^	Moderate	AUC-driven	APPROVED (restricted)	Maintenance-only; restricted indication; high variability accepted (Melmed et al., 2015)
MK-0616	Long**	Low	AUC-driven	NDA submitted	Macrocyclic PCSK9 inhibitor; C10 enhancer (Ballantyne et al., 2023)
I-338 insulin	∼60 h	Moderate	AUC-driven	Phase 2	Long t½ enables accumulation (Halberg et al., 2019)
Desmopressin (Minirin/Nocdurna)	∼3 h	Moderate	AUC	APPROVED (oral/SL)	Low dose; wide window
Liraglutide	∼13 h	Moderate	AUC/time-integrated	NOT FEASIBLE	Fails Gate 1: insufficient t½
Insulin (all analogs)	1–6 h	High	Cmax/peak-driven	NOT FEASIBLE	Fails Gates 1, 3, 4 (Cryer, 2008)
Glucagon	Minutes	High	Cmax/acute rescue	NOT FEASIBLE	Fails Gates 1, 3, 4
Calcitonin	∼1 h	Moderate	Mixed	FAILED (SMC021)	Fails Gate 1 (Karsdal et al., 2010)
PTH(1–34)	Minutes	High	Cmax-driven (pulsatile)	NOT FEASIBLE	Fails Gates 1, 4 (Neer et al., 2001)
Vasopressin analogs	10–35 min; ∼3 h (desmo)	High	Cmax/acute	LIMITED (sublingual)	Fails Gates 1, 3
Oxytocin	1–6 min	High	Cmax/acute uterotonic	NOT FEASIBLE	Fails Gates 1, 3, 4
Monoclonal antibodies	Days–weeks	Variable	AUC-driven	REDIRECT: Device	Fails Gate 5

^a^ Octreotide systemic t½ is short; the formulation uses immediate release with high concentration of peptide and enhancer. **MK-0616, pharmacokinetic data support a long effective half-life. Note: This analysis demonstrates that semaglutide occupies a unique position. The overwhelming majority of therapeutic peptides fail at Gate 1 or Gates 3–4.

**TABLE 3:** Regulatory outcomes of oral peptide programs.

Peptide/Program	Phase reached	Primary failure mode	Regulatory/Clinical issues	References
Oral Insulin (IN-105)	Phase III	Variability; bioequivalence failure	CV >100%; failed primary endpoint	Arbit and Kidron (2009)
Oral Insulin (ORMD-0801)	Phase III	Efficacy failure; variability	Failed HbA1c endpoint	Fonte et al. (2013)
Oral Calcitonin (SMC021)	Phase III	GI effects; regulatory rejection	EMA negative opinion; FDA CRL	Karsdal et al. (2010)
Oral PTH(1–31)	Phase II	Variability; inadequate profile	Unable to replicate pulsatile Cmax	Boyce et al. (2013)
Oral Growth Hormone	Phase I/II	Near-zero bioavailability	22 kDa protein too large	Rosenfeld and Hwa (2009)
Oral Octreotide (Mycapssa)	APPROVED (limited)	Partial success; high variability	Maintenance only; CV 50%–80%	Melmed et al. (2015)

This table summarizes documented regulatory outcomes illustrating the recurring failure modes predicted by the negative-selection framework.

This negative-selection framework is intentionally conservative. Its purpose is not to discourage innovation, but to channel it toward molecules whose biology permits success. By explicitly identifying what should not be attempted, the framework reduces the risk of repeating historical failures. It provides a rational foundation for decision-making in the development of oral peptides and proteins. Molecules that fail the framework should be directed toward the alternative routes discussed in Section 8 or the device-mediated approaches discussed in Section 9, rather than being abandoned entirely.

## 8 Route selection beyond oral: realistic alternatives for peptides and proteins

For peptides excluded from oral development by the negative-selection framework, the question becomes: if not oral, what then? This section surveys non-oral administration routes that may be appropriate for systemically acting peptides and proteins, applying the same pharmacokinetic constraints emphasized throughout this review. The goal is not to promote any particular route, but to provide a scientifically grounded framework for route selection that matches molecular properties to delivery feasibility.

### 8.1 Pulmonary/inhaled systemic delivery

The pulmonary route offers a large absorptive surface area (∼100 m2 in the alveolar region), a thin epithelial barrier (0.1–0.5 µm in alveoli), rich vascularization, and avoidance of first-pass hepatic metabolism. These features enable relatively rapid systemic absorption of appropriately formulated peptides, with bioavailability substantially higher than that of oral delivery, typically 10%–25% for peptides such as insulin, compared with <1% with oral delivery (Labiris and Dolovich, 2003; Patton et al., 2004). Recent reviews have comprehensively addressed emerging trends in pulmonary delivery of biopharmaceuticals, including novel formulation strategies and advances in devices (Onoue et al., 2022).

Weaknesses and limitations: Variability in absorption is heavily influenced by inhalation technique, device performance, and underlying lung pathology (e.g., asthma, COPD, smoking). Dose ceilings exist because only a limited amount of powder can be delivered per inhalation. Local tolerability concerns include cough, throat irritation, and theoretical risks of immunogenicity associated with repeated exposure to macromolecules. Device dependence introduces additional cost, training requirements, and potential adherence barriers (Ibrahim and Garcia-Contreras, 2015; Onoue et al., 2022).

Clinical experience: Inhaled insulin (Afrezza) demonstrates feasibility but also limitations: rapid onset suitable for mealtime use; bioavailability approximately 20%–25%; contraindicated in chronic lung disease and associated with cough in some patients. Earlier inhaled insulin products (Exubera) failed commercially despite regulatory approval, illustrating that technical feasibility does not guarantee market success.

Best fit: Potent peptides where rapid onset is desirable (mealtime insulin, rescue medications), dose is low enough to be delivered via inhalation, and the patient population does not have significant pulmonary comorbidity.

### 8.2 Nasal delivery (systemic or CNS-Targeted)

Nasal delivery offers noninvasive administration, relatively rapid absorption through the nasal mucosa (richly vascularized, thin epithelium), avoidance of GI degradation, and the potential for nose-to-brain delivery of CNS-targeted peptides via the olfactory and trigeminal nerve pathways.

Clinical experience: Nasal glucagon (Baqsimi) is feasible for rescue indications, with bioavailability of approximately 30%–40%, rapid onset suitable for hypoglycemia rescue, and needle-free administration important for caregivers treating unconscious patients (Rickels et al., 2016; Suico et al., 2018). As noted in recent clinical reviews, nasal glucagon represents a significant advance in hypoglycemia rescue. Nasal desmopressin has been used for years for diabetes insipidus and nocturnal enuresis. Other nasal peptide approvals include buserelin and nafarelin for endocrine indications, though not all remain widely marketed. Nasal calcitonin was approved for the treatment of osteoporosis, although market uptake was limited.

Best fit: Very potent peptides requiring small doses, rescue medications where needle-free administration is valuable (hypoglycemia, opioid overdose), or CNS-targeted strategies where direct nose-to-brain access may offer advantages over systemic administration.

### 8.3 Buccal/sublingual delivery

Buccal and sublingual delivery avoids GI degradation and partially avoids first-pass hepatic metabolism (venous drainage from these regions partially bypasses the portal circulation). Relatively rapid absorption through the oral mucosa. Good patient acceptability for appropriately formulated products (Caon et al., 2015; Hua, 2019).

Recent advances include the development of devices specifically designed to overcome the buccal mucosal barrier for peptide administration. Passive mucoadhesive films and patches have shown promise for specific peptides. Active device-based approaches, including microneedle patches, jet injectors, and ultrasound-enhanced delivery systems, are expanding the range of peptides amenable to this route (Malhotra et al., 2025; Karki et al., 2025). These technologies address the dose capacity and variability limitations of conventional buccal formulations. Oromucosal films incorporating permeation enhancers and optimized polymer matrices represent another promising direction for peptide delivery through the buccal route.

- Weaknesses and limitations: Small dose capacity is limited by dissolution rate and mucosal surface area. Saliva washout reduces bioavailability unless specialized formulations are used. Taste masking may be required. The bioavailability of peptides remains substantially lower than that of parenteral administration.
- Clinical experience: Sublingual desmopressin (Nocdurna) has been approved for the treatment of nocturia, and oral desmopressin (Minirin) remains available for diabetes insipidus. Various sublingual and buccal peptide formulations have been explored in clinical trials with mixed results.
- Best fit: Potent peptides requiring modest systemic exposure that can accommodate the variability inherent to this route. May be appropriate for peptides when oral is ineffective but pulmonary is unsuitable due to lung disease or other factors.

### 8.4 Transdermal and microneedle-assisted delivery

Classical transdermal delivery is generally not suitable for macromolecules due to the stratum corneum barrier, which limits passive diffusion to molecules smaller than approximately 500 Da with appropriate lipophilicity. Peptides and proteins exceed this limit by 1–3 orders of magnitude.

Microneedle patches (dissolving, coated, or hollow microneedles typically 300–800 um in length) penetrate the stratum corneum and deliver the drug into the viable epidermis or upper dermis. This effectively converts delivery to minimally invasive intradermal or subcutaneous dosing while maintaining patient acceptability superior to that of conventional hypodermic injection. However, current consensus suggests that microneedle approaches are more relevant for vaccines and immunotherapies (where skin immune cells are advantageous targets) than for chronic peptide therapy, which requires systemic exposure. Dose capacity limitations and the need for repeated application limit the practicality of most therapeutic peptides.

### 8.5 Long-acting injectables, depots, implants, and pumps

This category represents the most underutilized pragmatic alternative to oral delivery and should be considered the primary comparator against which oral approaches are evaluated. If the primary goal is to reduce injection burden rather than eliminate injections entirely, long-acting formulations often outperform oral approaches in terms of pharmacokinetic predictability, total cost, and clinical outcomes. For most peptides excluded from oral delivery by the negative-selection framework, long-acting injectable formulations represent the most practical, pharmacokinetically superior, and economically sustainable alternative.

Examples include once-weekly injectable semaglutide (Ozempic/Wegovy), which provides superior pharmacokinetic predictability compared to oral semaglutide at comparable efficacy; once-monthly dulaglutide and exenatide ER; and various long-acting GnRH agonists (Sartor, 2003). Extended-release formulations using microspheres, *in situ* gelling systems, and implantable devices can provide weeks to months of therapeutic coverage from a single administration.

The comparison between oral semaglutide and injectable semaglutide is instructive. While oral semaglutide offers needle-free convenience, injectable once-weekly semaglutide provides more predictable pharmacokinetics, does not require fasting, and achieves comparable clinical outcomes. For many patients, the reduction from daily oral administration with fasting requirements to weekly injection without dietary restrictions represents a net improvement in convenience and adherence.

Best fit: Any peptide or protein where adherence to frequent injection is problematic, where PK predictability is essential, and where formulation feasibility exists. This approach often provides superior pharmacokinetic control compared to variable oral absorption. For short-half-life peptides that fail the oral feasibility test, long-acting injectable formulations may be the most realistic pathway to reduced treatment burden (Table 4).

**TABLE 4:** Route triage matrix: Matching peptide/protein properties to delivery routes.

Molecular property	Oral	Pulmonary	Nasal	Microneedle	LAI/Depot
Short t1/2 (<24 h)	X Not feasible	+/− If rapid onset	+/− Rescue/acute	+/− If frequent OK	CHECK Extended release
Long t1/2 (>24 h)	CHECK If all criteria met	+/− Possible	+/− Possible	CHECK Good fit	CHECK Excellent fit
Narrow window	X Variability intolerable	X Variable	X Variable	CHECK Controlled	CHECK Predictable PK
Wide window	CHECK If t1/2 adequate	CHECK Good fit	CHECK Good fit	CHECK Good fit	CHECK Excellent fit
Peak-driven PD	X Cannot control timing	CHECK Rapid Cmax	CHECK Rapid Cmax	+/− Depends	X Sustained inappropriate
AUC-driven PD	CHECK If accumulation	+/− Possible	+/− Possible	CHECK Good fit	CHECK Excellent fit
Protein (MW > 5 kDa)	X No epithelial absorption	+/− Limited	X Not feasible	CHECK Bypasses barrier	CHECK Standard approach
Rescue/acute indication	X Onset slow/variable	CHECK Rapid onset	CHECK Rapid, needle-free	+/− Onset adequate	X Not for acute
Chronic daily therapy	+/− If boundary-case	+/− Device burden	+/− Tolerability	CHECK Good acceptance	CHECK Reduced frequency

CHECK, Well-suited; +/− = conditionally suitable; X = Generally not suitable. LAI, Long-acting injectable.

## 9 Swallowed device-mediated injection delivery: bypassing epithelial absorption

A fundamentally different approach to oral protein delivery has emerged in recent years: ingestible devices that bypass epithelial absorption entirely by delivering macromolecule payloads directly into tissue. Although these devices are swallowed and pass through the gastrointestinal tract, they deliver payloads via tissue injection rather than via epithelial absorption. The term swallowed injection distinguishes this mechanism from classical oral absorption while acknowledging the oral route of administration. These technologies do not solve oral absorption; they redefine swallowing as a route to device-mediated injection. Understanding this distinction is essential to avoid conflating device-enabled delivery with classical oral pharmacology.

### 9.1 Conceptual framing: swallowed injection, not oral absorption

Device-mediated delivery systems share a familiar premise: systemic protein delivery via classical oral absorption is untenable and must be circumvented. By abandoning epithelial absorption and substituting controlled tissue deposition, these technologies implicitly acknowledge the failure of classical oral pharmacology for proteins. They therefore constitute a distinct delivery modality governed by device performance, tissue safety, and combination-product regulation, rather than by improvements in bioavailability.

### 9.2 New constraints, unresolved questions, and critical evaluation

Device-mediated approaches introduce constraints distinct from those of classical oral formulation that warrant careful problematization:

Variability structure: Dose delivery becomes a binary or quasi-binary event, depending on actuation success. Either the device fires and delivers the payload, or it does not. This creates a different variability structure than that of conventional oral formulations, in which absorption varies continuously. For some applications, binary delivery may be preferable (apparent success/failure); for others, it may introduce new risks (complete dose failure if the device malfunctions).

Chronic tissue safety: Repeated penetration of gastrointestinal tissue raises unresolved questions regarding cumulative injury, ulceration, scarring, bleeding risk, or tissue remodeling. While single-dose or short-term studies may demonstrate acceptable safety, the long-term consequences of daily or weekly tissue penetration over years of chronic therapy remain to be characterized. This represents a significant unknown that peer-reviewed clinical data have not yet addressed.

Device retention and passage: The fate of device components after payload delivery must be carefully considered. Retained hardware may cause obstruction, perforation, or chronic irritation. Complete dissolution or reliable passage must be demonstrated across diverse patient populations with varying gastrointestinal anatomy and motility patterns.

Regulatory pathway: Evaluation shifts toward device reliability, failure modes, and combination-product regulation, a fundamentally different framework than drug-only approval. Sponsors must satisfy both drug and device regulatory requirements, potentially involving different review divisions and expertise.

Manufacturing economics: Production must absorb the cost of manufacturing both drug and device components. For high-value biologics (e.g., monoclonal antibodies costing thousands of dollars per dose) or indications in which injection avoidance offers exceptional benefit, these trade-offs may be acceptable. For commodity biologics or indications in which injection is well tolerated, the added complexity and cost may not be justified (Table 5).

**TABLE 5:** Device-mediated swallowed injection delivery systems: Current landscape.

Technology	Mechanism	Development stage	Key considerations	References
SOMA	Gastric self-orientation; spring-loaded needle	Academic proof-of-concept (swine)	Orientation reliability; chronic safety	Abramson et al. (2019)
Cephalopod microjet	High-velocity liquid jet into GI tissue	Academic demonstration	Jet penetration; payload stability	Arrick et al. (2024)
Robotic capsule/RaniPill-type	Intestinal microneedle deployment	First-in-human (company-reported)	Device reliability; combination-product regulation	Myers et al. (2024)
BIOND/Bioject system	Needle-free jet injection	Development	GI tissue adaptation; energy delivery	Company data
Ultrasound-enhanced delivery	Cavitation-mediated permeabilization	Preclinical	Energy delivery; tissue effects; localization	Various
Osmotic-driven injection capsules	Pressure-driven tissue injection	Development	Actuation reliability; payload capacity	Various
Ingestible electronics with reservoirs	Electronic-triggered release into tissue	Early development	Power; biocompatibility; passage	Various

The progress of commercial device programs should be evaluated based on peer-reviewed clinical data, when available. Device-mediated systems should be evaluated as injection delivery rather than oral absorption.

At present, ingestible injection and microjet systems represent the only credible pathways under development for the systemic delivery of proteins via a swallowed dosage form. Their progress should be evaluated on their own merits as device-enabled injection delivery, not as validation of oral protein absorption. Maintaining this distinction is essential to avoid repeating the conceptual errors that have driven a century of failed attempts to develop classical oral delivery.

## 10 Conclusions: route selection, not platform optimism

The aspiration to deliver peptides and proteins orally has endured for more than a century, driven by the undeniable practical advantages of oral administration and by successive waves of technological optimism. However, the accumulated evidence reviewed in this article leads to a clear and inescapable conclusion: for most peptides and essentially all proteins intended for systemic action, oral delivery through classical absorption-based mechanisms is not merely challenging but fundamentally incompatible with biological, pharmacokinetic, regulatory, and economic realities.

The approval of oral semaglutide represents a genuine scientific achievement, but its meaning has frequently been overstated. Semaglutide succeeded not because the field solved oral peptide delivery as a general problem. It succeeded because it possesses an extraordinary constellation of properties, an elimination half-life measured in days rather than hours, exceptional receptor potency, a wide therapeutic window, and pharmacodynamic effects driven by time-integrated exposure rather than peak concentration, which enable it to accommodate the severe constraints of oral administration. These features enable semaglutide to accumulate despite its low bioavailability and high interindividual variability in absorption. Most peptides do not share these properties, and no formulation strategy can confer them where they do not already exist.

Three distinct failure modes explain the historical record of oral peptide and protein failure: (1) Exposure infeasibility: A short elimination half-life prevents accumulation to a therapeutic body burden at low oral bioavailability, regardless of how sophisticated the formulation technology. (2) Variability-driven regulatory failure: Coefficients of variation exceeding 50%–60% preclude bioequivalence demonstration for dose-critical molecules whose therapeutic effects depend on achieving specific concentrations at specific times. (3) Dose escalation effects and economic impossibility: Compensating for poor absorption primarily increases luminal concentration rather than systemic exposure, causing gastrointestinal class effects and inflating manufacturing costs to prohibitive levels before achieving therapeutic efficacy.

This review has advanced a negative-selection framework as a necessary corrective to platform-driven extrapolation. Rather than asking how to make all peptides and proteins oral, development efforts should begin by identifying which molecules should not be pursued for oral delivery under any reasonable assumptions. Short-half-life peptides (<24 h), dose-critical hormones with narrow therapeutic windows, peak-dependent molecules requiring precise timing, and systemically acting proteins fall squarely within this exclusion category. By contrast, only a narrow class of peptides with long elimination half-lives, high potency, expansive therapeutic windows, and time-integrated pharmacodynamics occupies the boundary of feasibility exemplified by semaglutide.

For molecules excluded from oral development, this review has surveyed realistic alternatives that deserve systematic consideration: pulmonary delivery for rapid-onset needs, nasal delivery for rescue applications, buccal/sublingual delivery for potent peptides accommodating variability, and most importantly, long-acting injectables, depots, implants, and pumps that often outperform oral approaches on pharmacokinetic predictability and total cost. Device-mediated swallowed injection systems represent a distinct modality for proteins that cannot be absorbed through epithelial barriers; however, these should be evaluated as combination products governed by device reliability and tissue safety rather than as evidence of oral absorption.

While this review emphasizes biological constraints, ongoing advances in formulation science deserve acknowledgment. Optimized release of peptide-enhancer combinations from novel dosage forms, informed by an improved understanding of pH- and physicochemical-based interactions in simulated gastrointestinal environments, may expand the boundaries of oral feasibility. Such advances may enable oral delivery for peptides that currently fall just outside the feasibility boundary. Nevertheless, fundamental pharmacokinetic constraints related to half-life and accumulation will continue to govern which molecules can benefit from improved formulation, and the negative-selection framework remains applicable across delivery technologies.

The emergence of small-molecule GLP-1 agonists, such as orforglipron, which can be taken without fasting restrictions, may further reshape the competitive landscape. For GLP-1 receptor agonism specifically, the future may belong to small molecules that overcome the constraints of peptide delivery. This development reinforces the central message that success in oral delivery depends on molecular properties rather than delivery platforms.

Progress in peptide and protein therapeutics will ultimately depend not on overcoming biology through ever more complex delivery systems, but on aligning development strategies with biological constraints. Oral delivery should be pursued when pharmacology permits and abandoned when it does not. Recognizing this distinction is not a retreat from innovation; it is a prerequisite for sustainable, meaningful advancement in the field.
